# Potential value of neuroimmunotherapy for COVID-19: efficacies and mechanisms of vagus nerve stimulation, electroacupuncture, and cholinergic drugs

**DOI:** 10.3389/fimmu.2023.1197467

**Published:** 2023-07-05

**Authors:** Xianqiang Yu, Qingming Kong

**Affiliations:** ^1^ Women and Children's Hospital Affiliated to Qingdao University, Heart center, Qingdao, China; ^2^ University of California, Los Angeles, Department of Cardiology, Los Angeles, CA, United States; ^3^ School of Laboratory Medicine and Bioengineering, Key Laboratory of Biomarkers and In Vitro Diagnosis Translation of Zhejiang province, Key Laboratory of Bio-tech Vaccine of Zhejiang Province, Engineering Research Center of Novel Vaccine of Zhejiang Province, Hangzhou Medical College, Hangzhou, China

**Keywords:** COVID-19, SARS-CoV-2, neuroimmunotherapy, vagus nerve, electroacupuncture, cholinergic drugs

## Abstract

COVID-19 is an inflammatory disease with multiple organs involved, mainly respiratory symptoms. Although the majority of patients with COVID-19 present with a mild to moderate self-limited course of illness, about 5-10% of patients with inflammatory disorders in severe COVID-19 have life-threatening progression. With the exception of a few drugs that have shown outstanding anti-COVID-19 effects, the efficacy of most drugs remains controversial. An increasing number of animal and clinical studies have shown that neuromodulation has a significant effect on reducing inflammatory markers of COVID-19, thus exerting an effective neuroimmunotherapeutic value. Currently, the main neuroimmunomodulatory measures effective against COVID-19 include vagus nerve stimulation, electroacupuncture, and cholinergic drugs. In this review, we will summarize the research progress of potential value of this neuroimmunotherapy measures for COVID-19 and elaborate its efficacies and mechanisms, in order to provide reliable evidence for clinical intervention.

## 1 Introduction

The COVID-19 pandemic continues to spread in an unprecedented way, causing inconvenience and challenges around the world. In particular, asymptomatic infections can lead to rapid spread of the virus (1–3). Although the majority of cases are mild or moderate, about 5%-10% of cases with inflammatory disorders are severe and life-threatening. This means that in most cases COVID-19 patients do not need antiviral treatment. However, waiting until a patient is seriously ill to start treatment may miss the window for early treatment, during which the course of the disease is more likely to change. Therefore, it is urgent to explore effective interventional measures (4).

Since the outbreak of COVID-19 more than three years ago, the search for intervention strategies to prevent or combat novel coronavirus has become the focus of clinical research (5). It is generally considered that antiviral and non-antiviral therapy, inflammatory regulation, and critical supportive care constitute the entire COVID-19 treatment system. In particular, anti-inflammatory therapy is an important part of the whole treatment. As treatment protocols are constantly updated and optimized during the pandemic, clinicians are required to keep an eye on treatment plan for COVID-19. Moreover, vaccines are a routine medical strategy against the virus, and many countries have been preparing them since the beginning of the pandemic (6, 7). As a result, restarting antiviral vaccine research will take more time and cost. Although there are no specific drugs or treatments officially approved to treat COVID-19, some therapies and drugs have shown promising results in preclinical or clinical trials (8, 9). However, many factors such as technical requirements and virus variation have always restricted the clinical efficacy. At the same time, immediate validation of COVID-19 with known antiviral drugs is a highly effective screening route. The process of discovering a new drug typically takes a decade or more, which is clearly not appropriate for the current emergency outbreak. One effective method currently in use is to test whether existing drugs or methods have antiviral/anti-inflammatory activity against SARS-COV-2, and computer-aided and structure-based means design approaches play an important role in this process (10). Therefore, antiviral measures repurposing is currently the optimal choice for screening anti-COVID-19 methods due to its time-saving and labor-saving characteristics.

As a variety of existing neuromodulation methods, vagus nerve stimulation, electroacupuncture, and cholinergic drugs have been shown to have significant anti-inflammatory effects on a variety of inflammatory diseases (11–13). Especially since COVID-19 pandemic, it has been gradually applied to the anti-inflammatory regulation function after SARS-COV-2 infection, and has obtained obvious antiviral value. This further proves the important value of neuroimmunomodulatory in the fight against cytokine reaction due to SARS-COV-2. Therefore, we will comprehensively elaborate and sort out the treatment approaches in these three major neuroimmunomodulatory aspects, so as to provide clinicians with standards and guidance for the selection of measures against COVID-19.

## 2 Virology

Common symptoms reported for COVID-19 were dry cough, fever, fatigue and shortness of breath (14). In addition, multiple organ manifestations, mainly digestive symptoms, were gradually reported (15). Most COVID-19 cases have mild symptoms, but in some cases, cytokine storms can be triggered, followed by acute respiratory distress syndrome (ARDS), septic shock, and even multiple organ failure, leading to death (16). Therefore, it is particularly important to further clarify the characteristics of COVID-19 and explore effective interventions.

**Figure 1:**
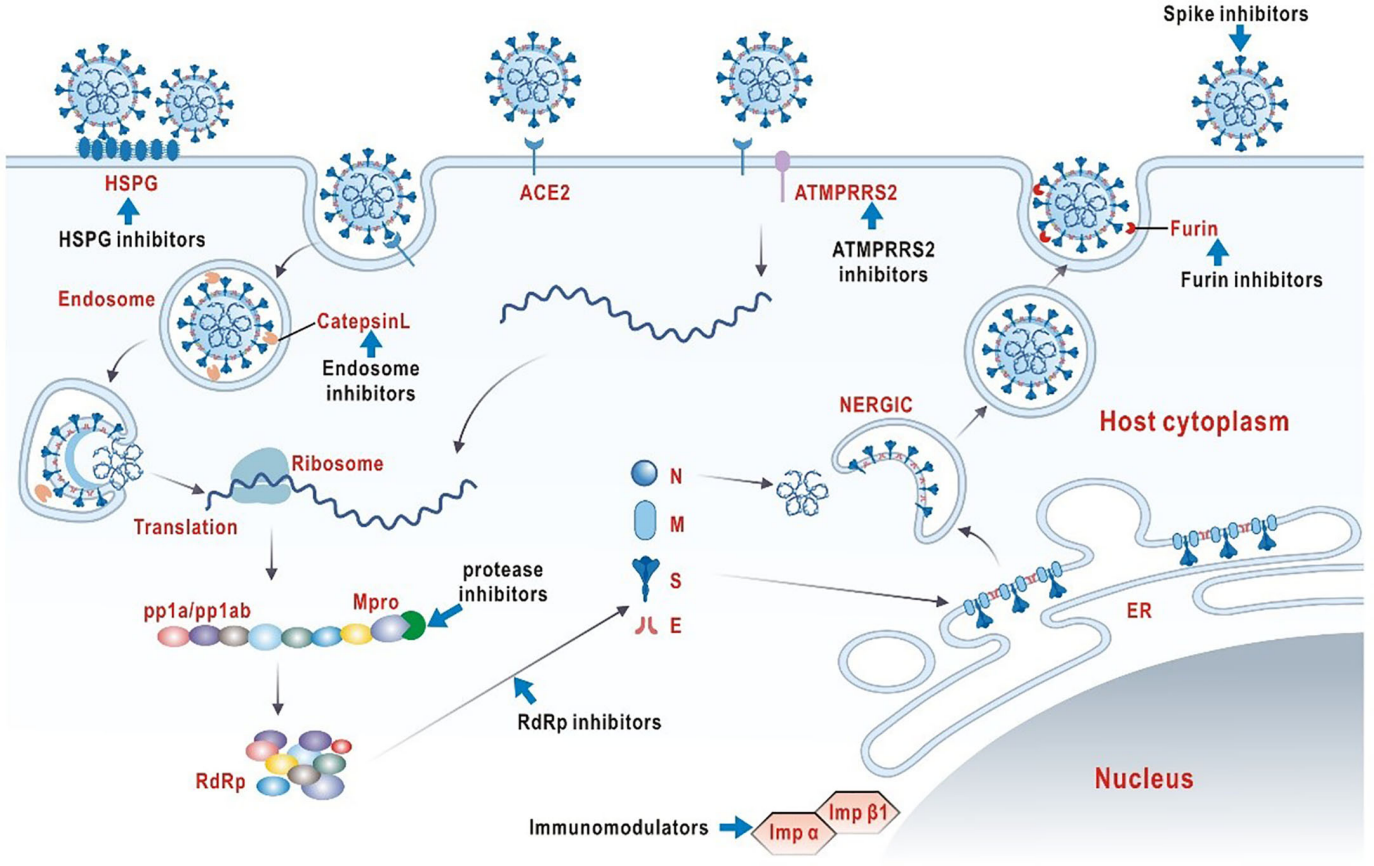
The structure of SARS-COV-2 and potential targets for intervention.

It is vital to clarify the structural and biological characteristics of SARS-COV-2 before exploring effective antiviral drugs (**Figure 1**). SARS-COV-2 is a member of the single-stranded RNA coronavirus family, as are previously known severe acute respiratory syndrome (SARS) and middle east respiratory syndrome (MERS) (17). The high diversity of SARS-COV-2 is attributed to its susceptibility to mutation and recombination. Humans, mammals and birds are the hosts of infection. In particular, novel coronavirus infection in humans mainly shows symptoms of multi-system infection mainly in respiratory tract (18). Coronavirus enters host cells *via* a trimer spike glycoprotein that gives the virus its coronavirus appearance, which is now recognized as the main mode of infection. It should be emphasized that SARS-COV-2 spikes have a stronger binding affinity for host receptors than other coronavirus, which may account for the relatively high rate of transmission and incidence of SARS-COV-2 (19, 20). The spike consists of two subunits, S1 and S2. The top of the S1 subunit, binds to the angiotensin converting enzyme 2 (ACE2) receptor on the surface of the host cell.S2 subunit fuses with the host cell membrane. When the S1 subunit binds to the receptor, the host trans-membrane serine protease 2 (TMPRSS2) activates the spikes and cleaves ACE2 by acting on the S2 subunit. This cleavage promotes the fusion of the virus with the cell membrane (21). Another infection mechanism is a PH-dependent endocytosis pathway (22). In summary, viral replication requires the help of different biological processes in the infected host cell, including endocytosis on the plasma membrane, uncoating and release of viral genomes into the cytoplasm, transcription and translation of viral RNA, and release of mature virions through golgi vesicles (23). In theory, blocking any infection process could have an antiviral effect.

At present, the selection and development of laboratory or clinical anti-SARS-COV-2 drugs are mainly based on the above understanding of the structure, the infection mechanisms of SARS-COV-2 and the clinical symptoms COVID-19 infection. Till now, antiviral therapy options are still very limited (24, 25). On the other hand, COVID-19 is a rapidly evolving inflammatory disease. In severe cases, uncontrolled inflammatory responses and cytokine storms are common (26). Therefore, regulating the inflammatory state is essential to improve the prognosis of severely ill COVID-19 patients. In this review, our goal is to systematically summarize the current effective means of neuro-immune regulation worldwide for COVID-19 to better facilitate clinical interventions and future antiviral strategies.

## 3 Clinically or laboratory effective neuroimmunotherapy for COVID-19

In epidemics of viral etiology, past experience suggests that early and indiscriminate use of neuroimmunotherapy is logical and beneficial for certain aggressive and rapidly mutating types. Therefore, this is also the empirical basis for the tentative application of existing means of neuro-immune regulation at the beginning of the COVID-19 outbreak. In this section we will list those anti-COVID-19 neuroimmunomodulatory tools that have shown some efficacy in clinical trials or that look promising in this regard.

### 3.1 Vagus nerve stimulation against COVID-19

COVID-19 is an infectious disease caused by SARS-CoV-2 invading cells through angiotensin 2 converting enzyme (ACE2) receptors that are heavily expressed in alveolar epithelial cells, which is how SARS-CoV-2 infects other organs (27). COVID-19 causes a storm of inflammatory factors (increased levels of interleukin-1β, IL-2, IL-6, IL-10, TNF, interferon, etc.), promoting the sympathetic-vagus balance disorder (1, 28). In turn, the body’s neural immunity regulates inflammatory states in response to infection (29). It is clear that nervous system immunity plays an important role in the inflammatory process after infection.

To the best of our knowledge, the immune system is mediated and regulated by afferent and efferent fibers of the vagus nerve, the main nerve of the parasympathetic nervous system (30). As a major component of the neuroendocrine-immune axis, the nerve is playing a crucial role in the response to infection (29). This axis is the three information systems of nerve, endocrine and immune system, which regulate the functions of various organs and systems through the connection of information (cytokines, hormones, chemical transmitters, etc.) and cooperate with each other, so as to achieve the coordination and unity of the overall functions. From an immunological perspective, the parasympathetic vagus nerve plays an anti-inflammatory role, while the sympathetic nervous system may have both pro-inflammatory and anti-inflammatory effects (29). In particular, the anti-inflammatory potential of the vagus nerve has been gradually validated in laboratory and clinical studies in recent years. The pathway of vagus nerve involved in immune reflex mainly includes hypothalamic-pituitary-adrenal axis anti-inflammatory pathway and cholinergic anti-inflammatory pathway (29). Therefore, vagus nerve stimulation to regulate parasympathetic nervous system, activate related anti-inflammatory pathways, and rebuild abnormal sympathetically vagus balance play a potential clinical value in the treatment of COVID-19.

The vagus nerve originates from the 10th cranial nerve and its branches are widely distributed and circulated in the neck, chest and abdomen (31). The extensive projection of the vagus nerve means that it is involved in many functions of the body’s autonomic nervous system (32). The vagus nerve connects specific sensors and effectors around it to the central nervous system. Vagus-mediated connections involve projection to the hypothalamus and cerebral cortex, thus allowing vagus regulation into the subcortical and cortical regions of the brain. Thus, signals generated by the vagus nerve have the potential to affect a wide range of functions, and thus overall body protection (32). Vagus nerve stimulation can be performed through both invasive and non-invasive methods (33). Invasive stimulation usually involves implantation of electrodes around the left cervical branch of the vagus nerve, but implantation risks and costs are high. Non-invasive stimulation in the outer ear using skin electrodes is easy to use. The afferent branch of the cervical vagus nerve can also be applied to the neck for non-invasive stimulation *via* the surface skin electrode of the handheld device (34).

Sensory fibers in the vagus nerve sense peripheral homeostasis (e.g. immune state) and respond accordingly (e.g. immune regulation). The auricular branch of the vagus nerve has its natural advantages as the preferred stimulation site for laboratory research or clinical application (35). The outer ear is the only peripheral branch of the vagus nerve from which it emanates. This area is susceptible to peripheral nerve stimulation.

With the COVID-19 pandemic, there is increasing laboratory or clinical evidence that auricular vagus nerve stimulation has a positive effect on COVID-19. It has been reported that stimulating auricular vagus nerve in severe COVID-19 patients can significantly reduce the levels of several pro-inflammatory mediators and increase the levels of related anti-inflammatory mediators (36). A similar study observed the same phenomenon, however, the overall clinical outcomes of COVID-19 patients treated with auricular vagus nerve stimulation did not improve (37). In addition, there have been cases where administering auricular vagus nerve stimulation to patients with COVID-19 significantly reduced blood levels of interleukin-6 (38). Animal experiments showed that auricular vagus nerve stimulation significantly restored endotoxin-induced tissue damage, and reduced the level of inflammatory cytokines and immune cells infiltrated by tissue, suggesting that the selection of auricular vagus nerve stimulation is of great significance as a feasible intervention for the treatment of acute inflammation (39). However, it has also been reported that although auricular vagus nerve stimulation can reduce some inflammatory indicators, it does not improve clinical symptoms (40). These studies have shown that vagus nerve stimulation has been used in treatment of cytokine reaction due to a virus and it was specific. Therefore, adequate basic experimental verification and randomized controlled studies are necessary to explore the role of auricular vagus nerve stimulation in COVID-19 regulation.

### 3.2 Electroacupuncture against COVID-19

To the best of our knowledge, COVID-19 is a respiratory infectious disease mainly with respiratory symptoms. However, other systemic symptoms, including gastrointestinal symptoms, accompany the disease process (18). We should accurately assess the potential threat of COVID-19 to the gastrointestinal (GI) tract and actively respond to it. Clinicians and scientists continue to try a variety of drug interventions, but their clinical effects and side effects have always affected the clinical treatment (5). However, to date there is still no clinically effective intervention for COVID-19. Therefore, the exploration and trial of effective and less invasive interventions will greatly improve the clinical course of patients with COVID-19. Acupuncture as a supplementary treatment is one of the fastest growing replacement therapy.

Acupuncture is a traditional Chinese medicine therapy with a long history in China, involves inserting fine needles into specific acupoints (41). The world health organization recommends acupuncture was applied to a wide range of diseases, including muscle bone disease, nervous system diseases, etc (42). According to traditional medical theory, acupuncture stimulation can promote the flow of qi, a life force that is said to circulate in the meridians known as meridians (43, 44). Acupoints are thought to be pathophysiological mechanisms associated with and possibly reflecting visceral and systemic conditions, and thus stimulation of specific acupoints may elicit responses that control the unbalanced internal environment and improve physical symptoms. Electroacupuncture (EA) is a modern modified acupuncture technique, which uses electric current to stimulate points rather than manual operation, and has the advantage of strong repeatability (45, 46). Over the past decade, several studies have explored the efficacy of EA in the treatment of functional gastrointestinal disorders, evaluating the effects of electricity on gastrointestinal secretory function, sensory, motor, and electromyographic activity (45, 47). To the best of our knowledge, EA is widely used in the treatment of various diseases due to its economical, reusable and low side effects. Some scholars used EA to stimulate the lung Shu point (BL13) to regulate inflammatory mediators, thus reducing the severity of viral pneumonia (48). Studies have also shown that electrical stimulation of Zusanli can significantly reduce abdominal pain and abdominal distension and other gastrointestinal symptoms caused by severe acute pancreatitis (49, 50). Therefore, these studies provide sufficient basis and reference value for EA to alleviate the gastrointestinal symptoms of COVID-19.

Currently, it is known that COVID-19 is mainly mediated by angiotensin converting enzyme II (ACE2) to infect cells, and the respiratory tract is the main target organ, while ACE2 is expressed to varying degrees in intestinal and gallbladder organs. This means a potential risk of infection of digestive tract organs (51). The experience of TCM in regulating gastrointestinal dysfunction suggests that electroacupuncture has potential clinical value in regulating gastrointestinal function of COVID-19.

Compared to respiratory symptoms, GI symptoms, although less common, have recently become more important (52). Studies shows that the GI tract symptoms can be as high as 50% (39.6 50%), including nausea (17.3%), diarrhea (12.9%), anorexia (12.2%), abdominal pain (5.8%), gas (5%) and vomiting (5%) (53, 54). The virus can also cause GI bleeding, acute pancreatitis, hepatitis, colitis and other GI diseases. Given the high rate of GI symptoms (diarrhea, nausea, etc.) in patients with COVID-19, screening these patients is critical. It is speculated that the virus can be re-transmitted through feces through aerosol containing virus droplets, which may constitute part of the cause of GI symptoms of COVID-19. However, this has not been confirmed (55). Based on this, sewage was evaluated to detect the presence of SARS-CoV-2 virus, thus identifying the occurrence of fecal-oral transmission and determining response strategies (56).

In addition to its high expression in the respiratory tract, ACE2 is highly expressed in GI cells, such as esophageal epithelial cells and absorptive enterocytes cells in the ileum and colon (57–59). SARS-CoV-2 virus can also enter the portal vein through the GI tract and affect vagus nerve function through vascular or lymphatic pathways. In addition, SARS-CoV-2 can further stimulate the central and peripheral nervous systems by binding cytokines, culminating in GI symptoms such as nausea (with or without vomiting).Therefore, the expression of ACE2 determines whether different organs are involved in the occurrence of symptoms and the degree of inflammation.

**Figure 2:**
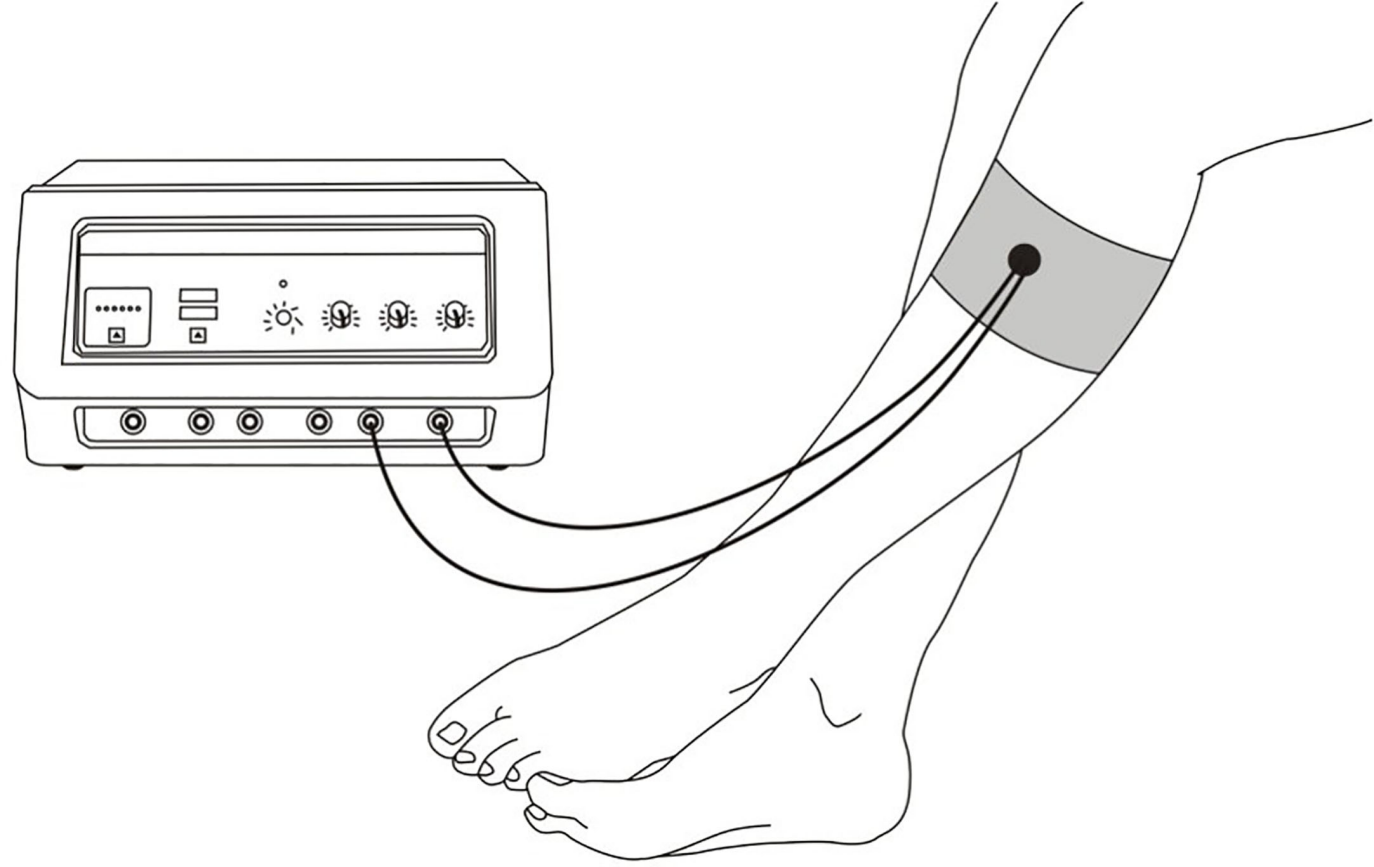
Schematic diagram of sites for regulating gastrointestinal function by electroacupuncture.

EA plays a role in regulating GI function by stimulating acupoints (**Figure 2**). Some scholars used EA to stimulate the lung Shu point (BL13) to regulate inflammatory mediators, thus reducing the severity of viral pneumonia (48). Studies have also shown that electrical stimulation of Zusanli can significantly reduce abdominal pain and abdominal distension and other GI symptoms caused by severe acute pancreatitis (49, 50, 60). Although the mechanism by which EA alleviates GI symptoms has not been fully elucidated, its clinical effect of significantly regulating inflammation and alleviating disease has potential value. Therefore, exploring the specific mechanism of EA regulating GI function will help to understand the principle of its function.

The esophagus is the passage that carries food to the stomach. At the gastroesophageal junction (GEJ), there is a thickened layer of muscle called the lower esophageal sphincter (LES). Esophageal motility abnormalities are classified into different types according to the function and contraction mode of LES (61). Recent studies have reported the effect of EA on esophageal dyskinesia.EA stimulation increases LES pressure (LESP) and peak peristalsis amplitude (62).

Gastric motility is one of the most important physiological functions of the human intestine. Without coordinated movement, digestion and absorption of dietary nutrients cannot occur. To perform its function efficiently, the intestine needs to produce not only simple contractions, but also coordinated contractions to produce transport (peristalsis) of luminal contents. Studies have shown that EA at ST36 restored the impaired gastric regulation induced by vagal nerve transection in dogs, but had no effect on gastric regulation in normal dogs (63). In addition, electrical stimulation significantly increased the number and amplitude of peak gastric EMG activity, suggesting increased gastric contractions after stimulation (64).

The small intestine moves in two different patterns: fasting and eating. The typical manifestation of fasting is the migration motor complex (MMC). Intestinal dyskinesia include loss of MMC, MMC damage. In experiments with rats, the investigators observed that EA at hind limb points (ST36 and SP6) significantly enhanced small intestinal transport (65).

There are staged contractions and large transitional contractions in the colon. Disrupted colonic motility is associated with a variety of functional disorders, such as irritable bowel syndrome (IBS), constipation, and diarrhea. EA stimulation increased colonic transport processes through the sacral parasympathetic efferent pathway (66). Similarly, EA stimulation of ST36 significantly increased contractility in the distal colon.

As for the pathophysiological mechanism of EA in regulating GI symptoms of COVID-19, we may find a speculative answer through a recent relevant study. In 2021, Ma et al. conducted an in-depth study on the neuroanatomy of EA stimulation therapy, and they confirmed that the physiological mechanism of electroacupuncture stimulation at ST36 site in mice is realized by driving the vagal adrenal axis (67). Therefore, EA has become an important method for neuroimmune regulation of COVID-19 to play an anti-inflammatory role. With the threat of infection from the ongoing spread of COVID-19, the effectiveness of EA in regulating the GI symptoms of COVID-19 is noteworthy.

### 3.3 Cholinergic drugs against COVID-19

With the COVID-19 pandemic, the cholinergic anti-inflammatory pathway (CAP), first articulated in 2003, has attracted widespread attention. Recent research into the pathogenesis of COVID-19 suggests that cholinergic anti-inflammatory systems may play a role. It is based on cholinergic neurotransmission and neuronal nicotinic acetylcholine receptor (nAChR). Acetylcholine (ACh) works by reducing pro-inflammatory cytokines and acts directly on the brain, released by cholinergic neurons to act on immune cells. It is important to emphasize that voluntary cholinergic neuron stimulation by nAChR activates skeletal muscle contraction.

The cholinergic system is primarily composed of nerve cells that communicate with other neurons and cells using or in response to the neurotransmitter Ach (68). Neuronal nAChR is a pentamer receptor-operated cationic channel composed of the is oplastid or heteromeric assembly of nine different isomers (69). Certain forms of nAChR can be activated by ACh. The homologous α7 receptor has been the focus of research in recent years. It is a high Ca2+ permeability nAChRs that responds to both choline and Ach (70). Studies in α7 nAChR ko mice have shown that responses to different inflammatory agents enhance tissue inflammation, suggesting that these receptors can negatively regulate inflammatory responses (71). The cholinergic regulation of macrophage function by nAChRα7 is a very extensive cholinergic anti-inflammatory pathway of vagus nerve in the regulation of tissue inflammation (72). Ach on macrophages exerts anti-inflammatory effects throughα7nAChR along the splenic nerve 9-11 *via* ganglia in the peritoneal - superior mesenteric plexus (73). α7nAChRs activation in macrophages down-regulates the production of pro-inflammatory cytokines through JAK2-STAT3 signaling pathway and blocking the activation of NF-κB pathway (74). Since the COVID-19 pandemic, studies have found that nicotine agonists play a key role in fighting the COVID-19 induced cytokine storm (75). Therefore, the cholinergic anti-inflammatory pathway, in which ACh is the key anti-inflammatory mediator.

The cholinergic anti-inflammatory pathway can be activated by external vagus nerve stimulation and drug intervention. One of the most hotly debated issues at the moment is the use of tobacco products (nicotine) and the risk of COVID-19 infection (76). The potential role of nicotine in COVID-19 pathology is that it itself may be used as an anti-inflammatory for COVID-19 through its interaction with the nicotine cholinergic system. Given its anti-inflammatory and potential role in interfering with the entry and/or replication of SARS-CoV-2, nicotine, nicotine agonists, or positive allosteric regulators of nicotine cholinergic receptors may play a therapeutic role in COVID-19. However, the negative effects of smoking on COVID-19 patients cannot be ignored, so a large number of studies are needed to prove it.

There is some preliminary evidence suggesting that the spike protein of SARS-CoV-2 interacts with human nicotinic acetylcholine receptors may have therapeutic potential for COVID-19 (77–79). As spike protein is similar to neurotoxins, it can bind to nAChR, including in the vagus nerve and in the brain (80). nAChRs are a type of receptor found on the surface of many different types of cells, including immune cells. They are involved in a wide range of physiological processes, including the regulation of the immune system, and their activation can lead to inflammation and other harmful effects (81). Therefore, if the spike protein does indeed bind to these receptors, it could potentially have a number of effects on the human body, including exacerbating the inflammatory response associated with COVID-19. Therefore, nicotinic receptors may affect the pathogenesis of SARS-CoV-2 infection. The dysregulation of nAChR by SARS-CoV-2 could promote a cholinergic dysfunction thus a central sympathetic drive leading to the cytokine storm.

Despite the potential harmful effects of this interaction, there is also some evidence to suggest that drugs that target nAChRs may have therapeutic potential for COVID-19. A drug called varenicline, which is used to treat nicotine addiction and also targets nAChRs, may have potential as a treatment for COVID-19 (82). The researchers found that varenicline was able to inhibit the replication of SARS-CoV-2 *in vitro*, and they suggested that it may have potential as a treatment for COVID-19.

Overall, while there is some evidence to suggest that the interaction between the spike protein of SARS-CoV-2 and human nAChRs may have therapeutic potential for COVID-19, more research is needed to fully understand the implications of this interaction and to develop effective and safe treatments.

## 4 Comparison of treatment plans

Each of the neuroimmunotherapy for COVID-19 has its own set of advantages and disadvantages. It is important now is that choosing appropriate treatment strategies depends on a better understanding of the pathophysiological background of COVID-19. We summarized some potential pros and cons of each treatment approach for better treatment decision making.

Vagus nerve stimulation has shown potential to reduce the cytokine storm and inflammation in severe cases of COVID-19. Non-invasive treatment that is relatively safe and well-tolerated, and can be administered through a wearable device that patients can use at home. Therefore, it could be assessed as a first resort early treatment for COVID-19 vulnerable population. On the contrary, cholinergic drugs, requiring a cardio-vascular monitoring, could be assessed as a therapeutic option for hospitalized patients. For instance, it could be interesting to evaluate whether the early treatment is sufficient to spare hospitalization or to improve the patient’s outcome thanks to an additive beneficial effect with cholinergic drugs used later in the course of the illness. However, the use of cholinergic drugs can have side effects, including nausea, vomiting, and diarrhea. Some cholinergic drugs can interact with other medications, making it important to monitor patients carefully. Electroacupuncture is also relatively safe and well-tolerated. It can be administered by licensed acupuncturists and can be incorporated into standard care. However, the use of needles may not be suitable for all patients, especially those who are immunocompromised or have bleeding disorders. The effectiveness of electroacupuncture may vary depending on the skill of the practitioner and the patient’s individual response.

In summary, while each treatment has its own advantages and disadvantages, more research is needed to fully understand their effectiveness and safety for treating COVID-19. It is important to consult with a healthcare professional to determine which treatment, if any, is appropriate for a particular patient.

## 5 Conclusion

The impact of neuro-immune regulation on COVID-19 is an area that should be studied in greater depth, as there may be an intersection of multiple pathophysiological processes. At present, neural immunity plays a key role in the process of COVID-19 and is also an important route for intervention. This evidence will guide further research, as there are many promising therapies that can prevent the SARS-CoV-2 virus. Vagus nerve stimulation effectively reduces inflammatory cytokine storm, providing a new strategy for the clinical treatment of COVID-19. Electroacupuncture, as an emerging neuroregulatory anti-inflammatory tool whose mechanism of action has just been elucidated, has a wide range of clinical applications including COVID-19 treatment. Cholinergic drugs, represented by nicotine agonists, can bind to nAChR and regulate inflammation by activating this receptor, bringing more thinking and challenges to COVID-19 prevention. What is important now is that choosing appropriate treatment strategies depends on a better understanding of the pathophysiological background of COVID-19, and neuro-immune regulation is a recurring theme in the growing body of scientific evidence.

## Author contributions

XY completed all of the content of the article, including analysis, writing and so on. QK is responsible for the overall design and review of the article.
